# Exosomal microRNA-503-3p derived from macrophages represses glycolysis and promotes mitochondrial oxidative phosphorylation in breast cancer cells by elevating DACT2

**DOI:** 10.1038/s41420-021-00492-2

**Published:** 2021-05-20

**Authors:** Shulin Huang, Peizhi Fan, Chaojie Zhang, Jing Xie, Xiaowen Gu, Shanshan Lei, Zihua Chen, Zhongcheng Huang

**Affiliations:** 1 The Hunan Provincial Key Lab of Precision Diagnosis and Treatment for Gastrointestinal Tumor, Xiangya Hospital, Central South University, Changsha, 410008 Hunan China; 2 Department of Breast and Thyroid Surgery, Hunan Provincial People’s Hospital (The First Affiliated Hospital of Hunan Normal University), Changsha, 410005 Hunan China; 3 Department of General Surgery, Hunan Provincial People’s Hospital (The First Affiliated Hospital of Hunan Normal University), Changsha, 410005 Hunan China

**Keywords:** Cell biology, Diseases

## Abstract

MicroRNAs (miRNAs) are emerging drivers in tumor progression, while the role of miR-503-3p in breast cancer (BC) remains largely unknown. We aimed to explore the impact of macrophage-derived exosomal miR-503-3p in the development of BC by regulating disheveled-associated binding antagonist of beta-catenin 2 (DACT2). miR-503-3p and DACT2 expression in BC tissues and cells was assessed, and the expression of Wnt/β-catenin signaling pathway-related proteins in BC cells was also evaluated. Macrophages were induced and exosomes were extracted. The screened BC cell lines were, respectively, treated with exosomes, miR-503-3p inhibitor/mimic or upregulated/inhibited DACT2, and then the phenotypes, glucose intake, oxygen consumption rate, and adenosine-triphosphate (ATP) level of BC cells were determined. Cell growth in vivo was also observed. MiR-503-3p was elevated, DACT2 was reduced, and Wnt/β-catenin signaling pathway was activated in BC cells. Macrophage-derived exosomes, upregulated miR-503-3p or inhibited DACT2 promoted malignant behaviors of BC cells, glucose intake, and activity of the Wnt/β-catenin signaling pathway, while repressed oxygen consumption rate and ATP level in BC cells. Reversely, reduced miR-503-3p or upregulated DACT2 exerted opposite effects. This study revealed that reduction of macrophage-derived exosomal miR-503-3p repressed glycolysis and promoted mitochondrial oxidative phosphorylation in BC by elevating DACT2 and inactivating Wnt/β-catenin signaling pathway. Our research may provide novel targets for BC treatment.

## Introduction

Currently, breast cancer (BC) is the most common malignancy in women and is a main cause of death. There were 1.6 million cases diagnosed with BC in the world every year^1^. Women living in low- and middle-income countries account for 53% of newly diagnosed BC cases and 62% of cancer-related death worldwide^2^. About 5–10% of all BC cases are resulted from genetic disorders, while 90–95% of cases are associated with environmental factors and life style^3^. Fortunately, in recent decades, the mortality of BC has been declined due to promoted awareness, advanced detection, and better treatments^4^. Patients with early and advanced-stage BC are treated with surgery combined with radiotherapy and chemotherapy. However, the prognosis of most patients to some chemotherapy drugs is poor because of multidrug resistance^5^. Hence, novel biomarkers of BC remain to be explored.

Exosomes derived from differentially activated macrophages have been reported to affect dormancy or resurgence of BC cells^6^. Exosomes can transport microRNAs (miRNAs) to promote intercellular communication and regulate immune response^7^. Exosomal miRNAs are more stable and exosomes transferring miRNAs contribute to the development of multiple tumor types^8^. For example, exosome-mediated miR-222 has been recorded to promote migration and invasion of BC cells^9^. MiR-503-3p is one of the miRNAs that has been verified to affect BC progression^10^. Nevertheless, the role of macrophage-derived exosomal miR-503-3p has not been investigated yet. Moreover, we found from the bioinformatic prediction that there were binding sites between miR-503-3p and disheveled-associated antagonist of β-catenin (DACT) 2, a member of the DACT family. DACT2 locus frequently harbors loss of heterozygosity in human cancers^11^ and DACT2 shows an association with BC development^12,13^. It has been implied that DACT2 could retard the processes of BC via inactivating Wnt/beta-catenin signaling^13^. Based on those reports, this research was performed to explore the impact of macrophage-derived exosomal miR-503-3p in biological processes of BC cells via modulating DACT2 and Wnt/β-catenin signaling pathway, and we inferred that the inhibition of macrophage-derived exosomal miR-503-3p may contribute to malignant behaviors of BC cells through targeting DACT2 and activating Wnt/β-catenin signaling pathway.

## Materials and methods

### Study subjects

One hundred and forty-one primary BC samples were collected from patients (26–80 years old, with a mean age of 51 years) in Hunan Provincial People’s Hospital between January 2012 and December 2015 and classified based on the 7th edition of Cancer Staging Manual proposed by the American Joint Committee on Cancer in 2010. According to the tumor, node and metastasis (TNM) stage, 26 cases were in stage І, 77 cases in stage ІI, 32 cases in stage ІII, and 6 cases in stage IV. Relative non-cancerous mammary epithelial tissues were harvested as the controls and stored in liquid nitrogen.

### Macrophage culture and induction

THP-1 has a monocyte phenotype which provides a tractable standardized substitute for human monocyte-derived macrophages. Human THP-1 cells were cultured with Roswell Park Memorial Institute (RPMI) 1640 medium supplemented with 10% fetal bovine serum (FBS). Then, cell suspension (10 μL) was centrifuged, and THP-1 cell pellets were resuspended in 10% FBS-RPMI 1640 medium, seeded into culture dishes at 1 × 10^6^ cells/well, and incubated with 200 nmol/mL propylene glycol methyl ether acetate (PMA) for 72 h. The adherent and polygonal cells were differentiated macrophages and were used for subsequent experiments^14^.

### Identification of macrophages

Macrophages were trypsinized, passaged, and incubated for 5 min. The cells were extracted, resuspended, and centrifuged. The sediment was added with fresh medium and cells were extracted and resuspended, and the cell suspension was used for counting. A total of 1 × 10^7^ cells were mixed, centrifuged, and then resuspended by phosphate-buffered saline (PBS), and this step was repeated three times. Cells were fixed with 4% formaldehyde and centrifuged, and the formaldehyde was removed. Centrifuged again, the cells were blocked with blocking buffer containing 5% bovine serum albumin (BSA) for 1 h and centrifuged, then appended with prediluted primary antibodies (anti-CD68, anti-CD204, and anti-CD206) at 4 °C for 1 h. Subsequently, the cells were centrifuged and incubated with secondary antibody for 45 min, then examined by a flow cytometer.

### Macrophage treatment and grouping

P4-P6 macrophages at 80% confluence were transfected with miR-503-3p inhibitor, miR-503-3p mimic, or the negative control (NC) (GenePharma Co., Ltd., Shanghai, China) based on lipofectamine^TM^ 2000 kit instruction (Invitrogen Inc., Carlsbad, CA, USA).

### Extraction of exosomes

PMA-stimulated macrophages were continuously incubated in 10% FBS-RPMI 1640 medium for 24 h. Exosomes were extracted using ultracentrifugation and macrophages in the culture flasks were cultured with serum-free medium for 24 h. Then, the supernatant was transferred into fresh tubes and the exosomes were purified. Supernatant was first centrifuged at 10,000 × *g* and 4 °C for 15 min, and filtered using a 0.22-μm filter (GSV Filtering Technology, USA). The Centricon Plus-70 centrifugal filtration unit (Millipore Inc., MA, USA) was used for exosome isolation, and the exosomes were stored at −80 °C^15^.

### Identification of exosomes

Transmission electron microscopy (TEM) observation: a TEM was used to observe the morphology and size of purified exosomes. The isolated exosomes were suspended in PBS (pH = 7.4), fixed with 2.5% glutaraldehyde, and transferred onto the formware/carbon-coated network (Iran Medical University, Tehran, Iran) for 20-min culture. Treated in 50 µL uranium oxalate (pH = 7, Merck, Darmstadt, Germany) for 5 min, the network was coated with methylcellulose/uranyl acetate (Merck) and put on ice for 10 min. Then, the redundant fluid was absorbed using the Whatman No. 1 filter paper and the network was air-dried for 5–10 min. The morphology and size of exosomes were observed under the Carl Zeiss Leo 906 TEM (Carl Zeiss, Oberkochen, Germany) at 80 kV, and the exosome size was confirmed according to the scale bar^15^.

Western blot analysis: CD63, tumor susceptibility gene 101 (TSG101), and CD81 (all 1:1000) were determined.

Nanoparticle tracking analysis (NTA): exosome samples were diluted with PBS at 1:7500 and 40 μL sample was appended into sample wells and conducted with compression (~700 Pa) to make the samples pass through the nanopores at uniform speed. The software Izon Control Suite 3.3.2.2000 was utilized to calibrate the data of samples and standards.

### BC cell screening and culture

Human normal mammary cell line HCF-10A and BC cell lines MDA-MB-231, CAL-51, HCC1937, SK-BR-3, and T47D (Cell Resource Center of Peking Union Medical College Hospital, Beijing, China) were incubated with Dulbecco’s modified Eagle medium (DMEM) containing 10% FBS, 0.1 U/L penicillin, and 100 mg/L streptomycin (Gibco, Carlsbad, California, USA), and medium was changed every 2 d. The cells were passaged and P2-P3 cells were collected. Reverse transcription-quantitative polymerase chain reaction (RT-qPCR) was used to determine miR-503-3p and DACT2 expression in the cell lines, and the protein expression of DACT2 was gauged using western blot analysis. Cell lines that had the largest and smallest difference in miR-503-3p and DACT2 expression from MCF-10A cells were screened for subsequent cellular experiments.

### Uptake of macrophage-derived exosomes

CAL-51 cells and MDA-MB-231 cells were seeded on the sterile slide at a density of about 30%. Then, FITC-miR-503-3p was transferred to M2 macrophages by electroporation, exosomes were extracted and stained with 10 μg/mL DIL staining agent for 30 min. BC cells were incubated for 48 h, and the co-localization of FITC and DIL was observed under a fluorescence microscope.

### Co-culture of exosomes and CAL-51 cells or MDA-MB-231 cells

Exosomes (20 μg) were resuspended and co-cultured with cells for 48 h for subsequent experiments.

CAL-51 cells were divided into eight groups: PBS group (cells were treated with PBS), exo group (cells were co-cultured with exosomes from untreated macrophages), miR-503-3p inhibitor-exo group (cells were co-cultured with exosomes from miR-503-3p inhibitor-transfected macrophages), exo + overexpressed (oe)-negative control (NC) group (macrophages were transfected with DACT2 overexpressed vector NC), exo + oe-DACT2 group (macrophages were transfected with DACT2 overexpression vector), inhibitor NC group (cells were transfected with miR-503-3p inhibitor NC), miR-503-3p inhibitor group (cells were transfected with miR-503-3p inhibitor), and miR-503-3p inhibitor + short hairpin RNA (sh)-DACT2 group (cells were transfected with miR-503-3p inhibitor and DACT2 silencing vector).

MDA-MB-231 cells were divided into PBS group (cells without any treatment), exo group (cells were co-cultured with exosomes from untreated macrophages), miR-503-3p mimic-exo group (cells were co-cultured with exosomes from miR-503-3p mimic-transfected macrophages), sh-NC-exo group (macrophages were transfected with DACT2 low expression vector NC), sh-DACT2-exo group (macrophages were transfected with DACT2 low expression vector), mimic NC group (macrophages were transfected with miR-503-3p mimic NC), miR-503-3p mimic group (macrophages were transfected with miR-503-3p mimic), and miR-503-3p mimic + Oe-DACT2 group (cells were transfected with miR-503-3p mimic and DACT2 overexpression vector).

### Detection of intracellular mitochondrial oxygen consumption rate

Cells were seeded onto 24-well plates of a Seahorse bioenergy detector (Seahorse Bioscience Inc, MA, USA) and incubated for 24 h. Subsequently, the cells were, respectively, appended with oligomycin (1.5 μmol/L), mitochondrial uncoupling agent carbonyl cyanide 4-(trifluoromethoxy) phenylhydrazone (1.0 μmol/L), antimycin (0.5 μmol/L), and rotenone (1.5 μmol/L). Then, the mitochondrial oxygen consumption rate was measured.

### Measurement of intracellular adenosine-triphosphate (ATP) level

The level of ATP in cells was determined by the ATPlite^TM^ luminescent analysis system (Perkin Elmer Inc., MA, USA). ATP level in the PBS group was defined as 100% and the relative ATP level in the experimental group was calculated. The relative ATP level (%) = (fluorescence intensity of the experimental group/fluorescence intensity of the PBS group) × 100%.

### Determination of glucose absorption level

The glucose absorption level was detected using glucose detection kits (Beijing Strong Biotechnologies, Inc., Beijing, China) (glucose oxidase assay). The cells were added with 198 μL detection reagent and 2 μL cell culture supernatant for 10-min incubation. The absorbance at 490 nm was analyzed by a microplate reader. The reagent blank and glucose content of empty wells of unseeded cells were set as the basic value, and the relative glucose absorption dose of cells in each well was calculated.

### Cell counting kit-8 (CCK-8) assay

According to the instruction of CCK-8 kit (Dojindo Laboratories, Kumamoto, Japan), CAL-51 cells or MDA-MB-231 cells (5 × 10^4^ cells/mL) were seeded at 6-well plates at 100 μL/well and incubated for 24, 48, and 72 h. Each well was supplemented with 10 μL CCK-8 solution and incubated for 3 h. The absorbance at 450 nm was assessed on a microplate reader.

### Propidium iodide (PI) single staining

CAL-51 cells or MDA-MB-231 cells were seeded into 12-well plates and adhered, then were starved by serum-free medium. Cells were trypsinized, fixed with 70% ethanol overnight, and incubated with RNaseA (prepared by PBS containing 0.2% Triton-X100, final concentration at 20 mg/L) for 30 min. Afterward, the cells were incubated with PI (50 mg/L) for 5 min and detected by a flow cytometer, then 20,000 cells were collected and analyzed by the ModFit software.

### Transwell assay

CAL-51 cells or MDA-MB-231 cells were made into cell suspension (3 × 10^5^ cells/mL) and added into the Transwell apical chambers (Corning, Tewksbury, MA, USA) coated with serum-free medium-diluted Matrigel (Matrigel was not added in the migration assay). Cells were incubated with relative reagent for 36 h and cells on filter membranes were removed. Next, the cells were fixed with pre-cooled (4 °C) 4% paraformaldehyde (Santa Cruz Biotechnology, CA, USA) and stained with crystal violet dye solution. Five fields of each membrane were selected and the number of transmembrane cells was counted under a light microscope.

### RT-qPCR

Trizol kit (TaKaRa, Liaoning, China) was used to extract the total RNAs in tissue samples or cells. The reverse transcription was performed using Mir-X miRNA kit and PrimeScript RT Master Mix (TaKaRa), and the PCR was conducted using SYBR Premix Ex Taq (Roche, CA, USA) to detect the relative expression of miR-503-3p and DACT2. The primers (Sangon, Shanghai, China) were shown in Table 1 and data were analyzed by 2^−ΔΔCt^ method. U6 and β-actin were used as the internal reference of miR-503-3p and DACT2, respectively^16^.

**Table 1:** Primer sequence.

Gene	Sequence
miR-503-3p	F: 5′-GGGGUAUUGUUUCCGCUGCCAGG-3′
	R: 5′-GGGCAGGGTCCGAGGT-3′
U6	F: 5′-CGCTTCGGCAGCACATATAC-3′
	R: 5′-TTCACGAATTTGCGTGTCAT-3′
DACT2	F: 5′-GGCTGAGACAACAGGACATCG-3′
	R: 5′-GACCGTCGCTCATCTCGTAAAA-3′
β-actin	F: 5′-TGGACATCCGCAAAGACCTGT-3′
	R: 5′-CACACGGAGTACTTGCGCTCA-3′

*F* forward, *R* reverse, *miR-503-3p* microRNA-503-3p, *DACT2* disheveled-associated binding antagonist of beta-catenin 2.

### Western blot analysis

Total protein in tissues and cells was extracted and protein concentration was determined via bicinchoninic acid kits (BOSTER Biological Technology Co., Ltd., Hubei, China). The extracted proteins were conducted with 10% sodium dodecyl sulfate-polyacrylamide gel electrophoresis (BOSTER Biological Technology) and transferred onto polyvinylidene fluoride membranes, which were blocked with 5% bovine serum albumin for 1 h. Then, the membranes were incubated with primary antibodies DACT2 (1:1000), active-β-catenin (1:5000), Glut1 (1:1000), β-actin (1:1000, all from Abcam Inc., MA, USA), p-β-catenin (1:1000), and lactate dehydrogenase A (LDH-A, 1:1000, both from Cell Signaling Technology, MA, USA) at 4 °C overnight. Next, the membranes were incubated with relative secondary antibody (Shanghai Miaotong Biotech Co., Ltd., Shanghai, China) for 1 h and developed using enhanced chemiluminescent reagent and the Bio-Rad Gel Doc EZ imager (Bio-Rad Laboratories, Hercules, CA, USA). Image J software (National Institutes of Health, Bethesda, Maryland, USA) was employed to analyze the gray values of the protein bands.

### Dual-luciferase reporter gene assay

Target relation between miR-503-3p and DACT2, and the binding sites of miR-503-3p and DACT2 were predicted at https://cm.jefferson.edu/rna22/Precomputed/. DACT2 3′-untranslated region (3′UTR) promoter sequence containing binding site of miR-503-3p was synthesized, and the DACT2 3′UTR wild type (WT) plasmid (DACT2-WT) and DACT2 3′UTR mutant type (MUT) plasmid (DACT2-MUT) were established. CAL-51 cells were co-transfected with DACT2-WT or DACT2-MUT, and mimic NC or miR-503-3p mimic. The relative luciferase activity was determined.

### Subcutaneous tumorigenesis in nude mice

Balb/C nude mice aged 4 w (Hunan SJA Laboratory Animal Co., Ltd., Hunan, China) were fed under the specific pathogen-free condition with 26–28 °C, 40–60% humidity, 12 h day/night cycle, and sterile food and water.

The nude mice were subcutaneously injected with 0.1 mL CAL-51 cells or MDA-MB-231 cells (2 × 10^6^), and the grouping was in line with the cell grouping (*n* = 6). The spirit, diet, defecation, and activity of the mice were observed. From the 4th day onward, the length-diameter (*a*) and width-diameter (*b*) were measured every 6 d. Tumor volume = 0.5 × *a* × *b*^2^. The tumor growth was observed and mice were euthanized by carbon dioxide 4 w later. The xenografts were collected and weighed.

### Statistical analysis

All data analyses were conducted using SPSS 21.0 software (IBM Corp., Armonk, NY, USA). miRNA and DACT2 expression in clinical samples were detected using paired double-sample Student’s *t* test. Data of groups were compared using Student’s *t* test or one-way analysis of variance (ANOVA) and were expressed as mean ± standard deviation. The correlation between miR-503-3p/DACT2 expression and clinical parameters of patients was analyzed using chi-square test. *P* value < 0.05 was indicative of statistically significant difference.

## Results

### MiR-503-3p is upregulated while DACT2 is downregulated in BC tissues and cell lines

**Fig. 1:**
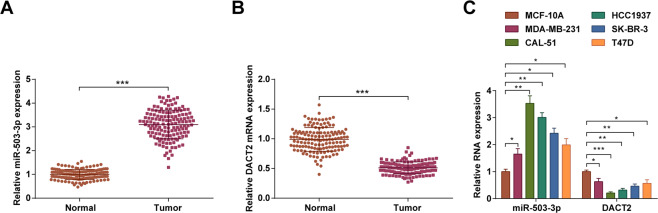
MiR-503-3p is upregulated while DACT2 is downregulated in BC tissues and cell lines. **A**, **B** RT-qPCR measured miR-503-3p and DACT2 mRNA expression in clinical samples, *n* = 141; **C** RT-qPCR measured miR-503-3p and DACT2 expression in BC cell lines and MCF-10A cells, repetitions = 3; *P < 0.05; **P < 0.01; ***P < 0.001; miR-503-3p and DACT2 expression in clinical samples were detected using paired double-sample Student’s *t* test and data of groups were compared using Student’s *t* test.

MiR-503-3p and DACT2 expression in BC and normal tissues were determined using RT-qPCR and it came out that (Fig. 1A, B) miR-503-3p expression was increased while DACT2 mRNA expression was decreased in BC tissues versus the normal mammary epithelial tissues (both *P* < 0.05).

miR-503-3p and DACT2 expression were also tested in cells and the results showed that (Fig. 1C) the BC cell lines (MDA-MB-231, CAL-51, HCC1937, SK-BR-3, and T47D) had higher miR-503-3p expression and lower DACT2 expression versus MCF-10A cells (both *P* < 0.05).

### Relationship between miR-503-3p expression and clinicopathological characteristics of BC patients

BC patients were classified into the high and low expression groups based on the median of miR-503-3p relative expression, and then the relation between miR-503-3p expression and clinicopathological characteristics of BC patients was analyzed. We found that (Table 2) patients with larger tumor, lymph node metastasis (LNM), and advanced TNM stage had an increased ratio of high expression of miR-503-3p, indicating that miR-503-3p expression was related to tumor diameter, LNM, and TNM (all *P* < 0.05), while not to age, menopause and histologic type (all *P* > 0.05).

**Table 2:** Relation between miR-503-3p expression and clinicopathological characteristics of BC patients.

Clinicopathological features	*n*	miR-503-3p expression		*P*
		High expression (*n* = 70)	Low expression (*n* = 71)	
Age (year)				0.179
≥51	68	38	30	
<51	73	32	41	
Menopause				0.375
No	47	26	21	
Yes	94	44	50	
LNM				0.001
No	78	29	49	
Yes	63	41	22	
Tumor diameter				0.004
<2 cm	60	21	39	
≥2 cm	81	49	32	
Histological classification				0.602
I–II	89	46	43	
III	52	24	28	
TNM stage				0.023
I–II	103	45	58	
III–IV	38	25	13	
Histological type				0.377
Infiltrative ductal carcinoma	73	40	33	
Infiltrative lobular carcinoma	45	21	24	
Other	23	9	14	

The data were enumeration data and analyzed by chi-square test.

*BC* breast cancer, *miR-503-3p* microRNA-503-3p, *TNM* tumor, node and metastasis, *LNM* lymph node metastasis.

### DACT2 is a direct target of miR-503-3p

**Fig. 2:**
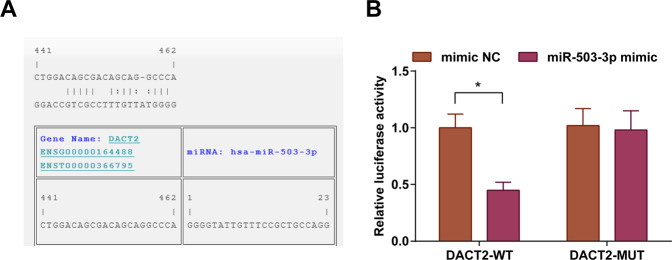
DACT2 is a direct target gene of miR-503-3p. **A** Online binding sites of miR-503-3p and DACT2; **B** dual-luciferase reporter gene assay verified the target relation between miR-503-3p and DACT2; *P < 0.05; the measurement data were expressed as mean ± standard deviation and compared using Student’s *t* test.

A target relation between miR-503-3p and DACT2 was predicted by a bioinformatic software (Fig. 2A). Outcomes of dual-luciferase reporter gene assay reflected that (Fig. 2B) cells that had been co-transfected with DACT2-WT and miR-503-3p mimic had a decreased luciferase activity (*P* < 0.05); the co-transfection of DACT2-MUT and miR-503-3p mimic did not influence the luciferase activity (*P* > 0.05), indicating that DACT2 was a direct target of miR-503-3p.

### MiR-503-3p/DACT2 axis regulates the Wnt/β-catenin signaling pathway, glycolysis, and mitochondrial oxidative phosphorylation (OXPHOS) in BC cells

**Fig. 3:**
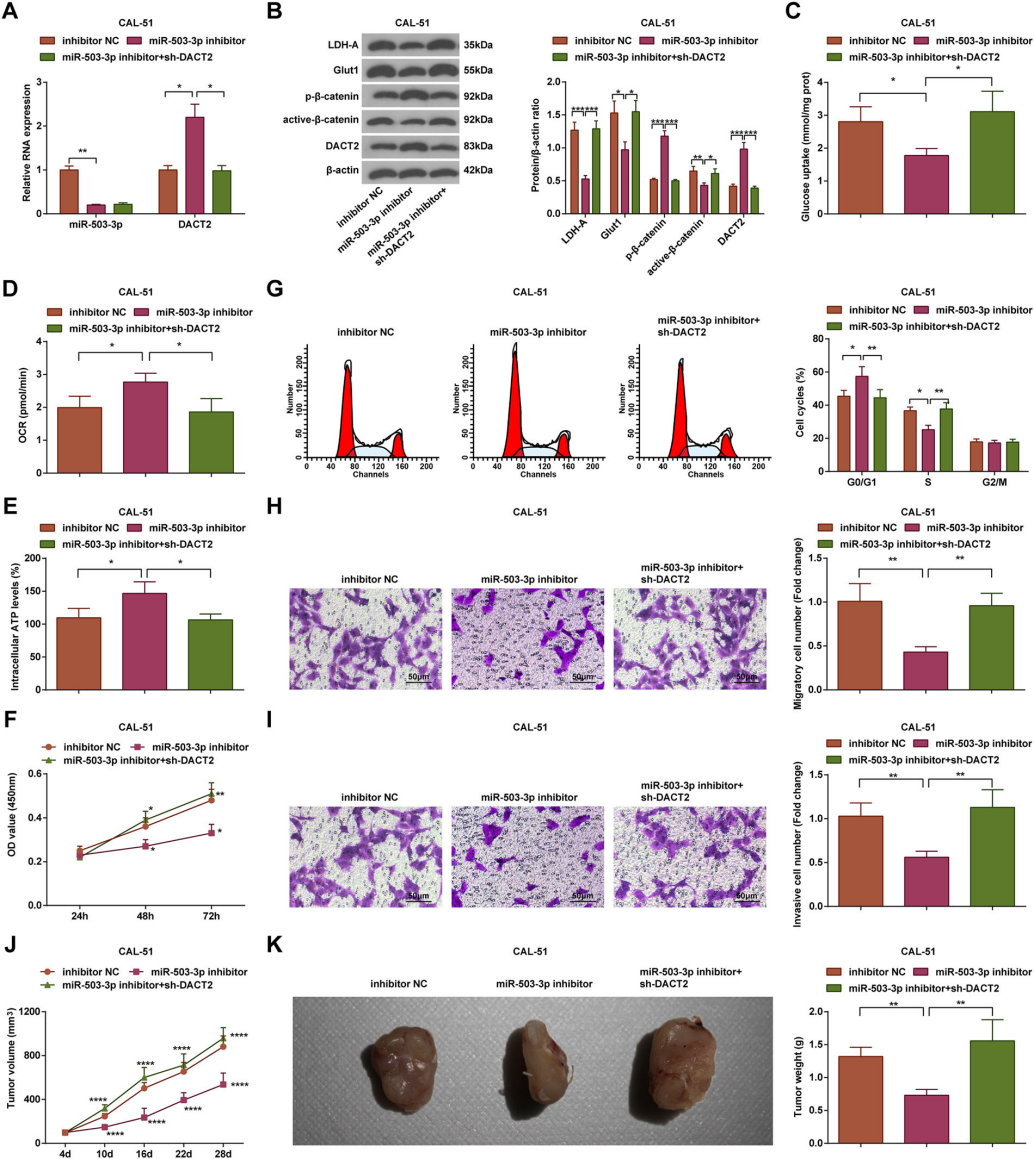
MiR-503-3p/DACT2 axis regulates the Wnt/β-catenin signaling pathway, glycolysis, and mitochondrial OXPHOS in BC cells. **A** RT-qPCR measured miR-503-3p and DACT2 expression in CAL-51 cells; **B** western blot measured DACT2, active-β-catenin, p-β-catenin, Glut1, and LDH-A protein expression in CAL-51 cells; **C** glucose intake of CAL-51 cells, **D** oxygen consumption rate of CAL-51 cells; **E** ATP level in CAL-51 cells; **F** CAL-51 cell viability; **G** flow cytometry determined cell cycle distribution. **H**, **I** Transwell assay tested migration and invasion ability of CAL-51 cells; **J**, **K** tumor volume and weight of nude mice that had been injected with CAL-51 cells; repetitions = 3 in (**A**–**I**), *n* = 6 in (**J**, **K**); *P < 0.05; **P < 0.01; ***P < 0.001; ****P < 0.0001; the measurement data were expressed as mean ± standard deviation and compared using Student’s *t* test.

Expression of miR-503-3p, DACT2, active-β-catenin, p-β-catenin, Glut1, and LDH-A in CAL-51 cells was evaluated. We observed that (Fig. 3A, B) relative to the inhibitor NC group, the miR-503-3p inhibitor group showed lower expression levels of miR-503-3p, active-β-catenin, Glut1, and LDH-A, and higher expression levels of DACT2 and p-β-catenin (all *P* < 0.05); relative to the miR-503-3p inhibitor group, DACT2 and p-β-catenin were downregulated, but active-β-catenin, Glut1, and LDH-A were upregulated in the miR-503-3p inhibitor + sh-DACT2 group (all *P* < 0.05).

We detected the intake of glucose (Fig. 3C), oxygen consumption rate (Fig. 3D), and ATP level (Fig. 3E), and noticed that versus the inhibitor NC group, the glucose intake was repressed while oxygen consumption rate and ATP level were increased in the miR-503-3p inhibitor group; relative to the miR-503-3p inhibitor group, the glucose intake was enhanced while oxygen consumption rate and ATP level were decreased in the miR-503-3p inhibitor + sh-DACT2 group (all *P* < 0.05).

The results of CCK-8 assay, Transwell assay, and flow cytometry indicated that (Fig. 3F–I) contrasted to the inhibitor NC group, the cell viability, migration, and invasion rates were inhibited, cell ratio in S phase was decreased while that in G0/G1 phase was increased in the miR-503-3p inhibitor group; versus the miR-503-3p inhibitor group, the cell viability, migration, and invasion rates were facilitated, cell ratio in S phase was increased while that in G0/G1 phase was decreased in the miR-503-3p inhibitor + sh-DACT2 group (all *P* < 0.05).

Results of subcutaneous tumorigenesis implied that (Fig. 3J, K) the tumor weight and volume were suppressed in the miR-503-3p inhibitor group versus the inhibitor NC group; in relation to the miR-503-3p inhibitor group, the tumor weight and volume were enhanced in the miR-503-3p inhibitor + sh-DACT2 group (*P* < 0.05).

In MDA-MB-231 cells, it was found that versus the mimic NC group, the miR-503-3p mimic group had higher miR-503-3p, active-β-catenin, Glut1, and LDH-A, as well as lower DACT2 and p-β-catenin expression levels (all *P* < 0.05). Relative to the miR-503-3p mimic group, reduced active-β-catenin, Glut1, and LDH-A, and increased DACT2 and p-β-catenin levels were measured in the miR-503-3p mimic + Oe-DACT2 group (all *P* < 0.05) (Supplementary Fig. 1A, B). Detection of intake of glucose, oxygen consumption rate, and ATP level revealed that versus the mimic NC group, the miR-503-3p mimic group showed enhanced intake of glucose, reduced oxygen consumption rate, and ATP level (all *P* < 0.05). In contrast to the miR-503-3p mimic group, the miR-503-3p mimic + Oe-DACT2 group demonstrated suppressed intake of glucose, and promoted oxygen consumption rate and ATP level (all *P* < 0.05) (Supplementary Fig. 1C-E). Moreover, observation of cell proliferation, cell cycle, invasion, and migration suggested that with respect to the mimic NC group, the cell viability, migration, and invasion rates were facilitated, cell ratio in S phase was increased while that in G0/G1 phase was decreased in the miR-503-3p mimic group (all *P* < 0.05). Versus the miR-503-3p mimic group, the cell proliferation, migration, and invasion rates were inhibited, cell ratio in S phase was decreased while that in G0/G1 phase was increased in the miR-503-3p mimic + Oe-DACT2 group (all *P* < 0.05). (Supplementary Fig. 1F-I). In tumor xenografts assay, increased tumor volume and weight were detected in the miR-503-3p mimic group rather than the mimic NC group; by comparison with the miR-503-3p mimic group, the miR-503-3p mimic + Oe-DACT2 groups manifested reduced tumor growth (both *P* < 0.05) (Supplementary Fig. 1J/K).

### Identification of macrophages and exosomes

**Fig. 4:**
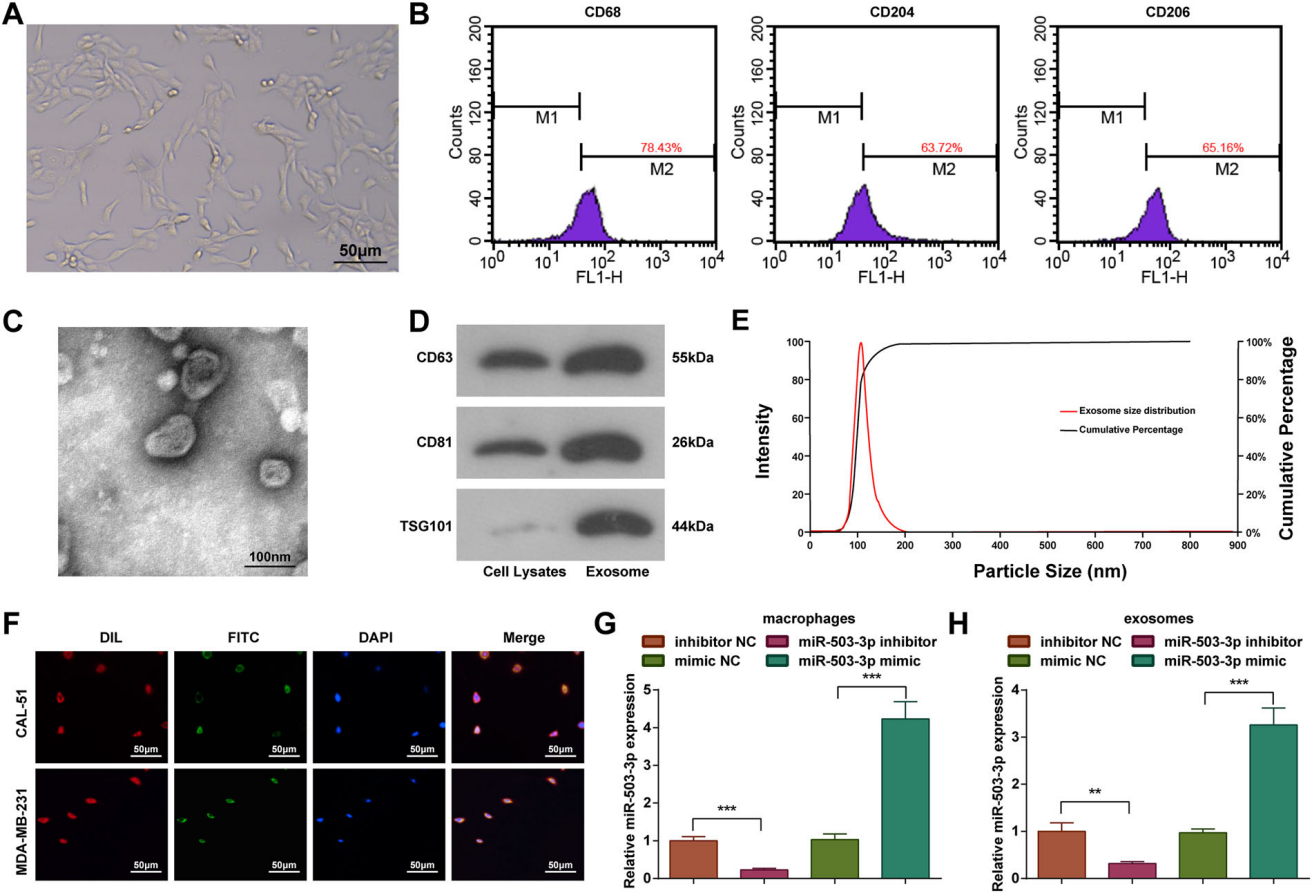
Identification of macrophages and exosomes. **A** Morphology of PMA-induced macrophages; **B** identification of macrophages by flow cytometry; **C** identification of exosomes under a TEM; **D** western blot measured CD63, CD81, and TSG101 levels; **E** NTA measured size and concentration of exosomes; **F** FITC and DIL staining assessed the activity of exosomes entering cells; **G**, **H** RT-qPCR measured miR-503-3p expression in macrophages (**G**) and exosomes (**H**); repetitions = 3; **P < 0.01; ***P < 0.001; the measurement data were expressed as mean ± standard deviation and compared using Student’s *t* test.

It was observed under a light microscope that (Fig. 4A) THP-1 cells were adherent after induced by PMA and IL-4, then became agglomerate and irregular. Subsequently, the cells extended pseudopodia and differentiated into adherent cells.

It has been reported that M2 macrophages were recruited by tumor cells. M2 macrophages-secreted chitinase 3-like 1 (CHI3L1), which could mediate mitogen-activated protein kinase pathway (MAPK) signaling to promote migration of gastric cancer and BC cells^17^. The positive expression of markers CD68, CD204, and CD206 on surface of adherent cells was determined by a flow cytometer, and we observed that (Fig. 4B) the expression rate of CD68, CD204, and CD206 was 78.43%, 63.72%, and 65.16%, respectively, which were in line with the characteristic phenotype of macrophages^18,19^.

The exosomes were extracted and identified by TEM observation, western blot analysis, and NTA. Under a TEM, there were round or oval membranous vesicles with complete envelope, typical cuplike structure and even size; membranous structure was observed in vesicle periphery and contained low-density substance with significant heterogeneity; the diameter of exosomes ranged from 30 to 100 nm (Fig. 4C). It was observed through western blot analysis that the surface markers of exosomes CD63, CD81, and TSG101 performed positive expression (Fig. 4D). The exosomes were diluted 7500 times by PBS and determined by NTA. The results indicated that the peak value of particle diameter of exosome was 106 nm and the particle concentration was 8.7 × 10^12^ particles/L (Fig. 4E).

In order to study that exosomes and miR-503-3p entered into cells, CAL-51 cells and MDA-MB-231 cells were seeded on a sterile slide of a well plate at a density of 30%. The FITC-miR-503-3p was electroporated into M2 macrophages, the exosomes were extracted, and the cells were incubated with 10 μg/mL DIL staining agent and observed under a fluorescence microscope. The co-localization of FITC and DIL in somatic cells indicated that the cells internalized exosomes containing FITC-miR-503-3p (Fig. 4F).

MiR-503-3p expression in macrophages and exosomes was determined using RT-qPCR and we found that versus the mimic NC group, miR-503-3p expression was increased in the miR-503-3p mimic group; contrasted to the inhibitor NC group, miR-503-3p expression was decreased in the miR-503-3p inhibitor group (*P* < 0.05) (Fig. 4G, H).

### Macrophage-derived exosomal miR-503-3p activates the Wnt/β-catenin signaling pathway, promotes glycolysis, and reduces mitochondrial OXPHOS in BC cells

**Fig. 5:**
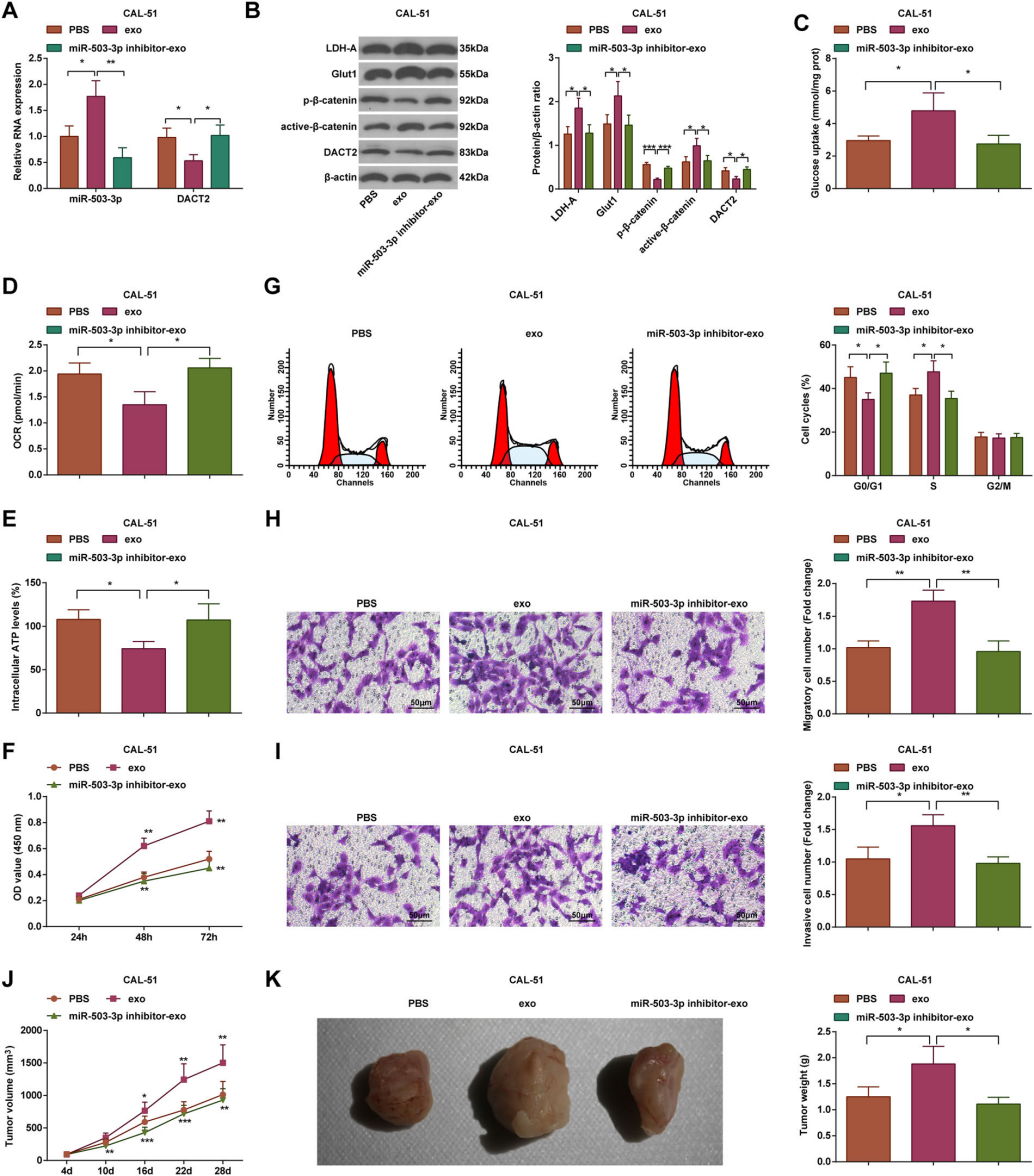
Macrophage-derived exosomal miR-503-3p activates the Wnt/β-catenin signaling pathway, promotes glycolysis, and reduces mitochondrial OXPHOS in BC cells. **A** RT-qPCR measured miR-503-3p and DACT2 expression in CAL-51 cells; **B** western blot analyzed DACT2, active-β-catenin, p-β-catenin, Glut1, and LDH-A protein expression in CAL-51 cells; **C** glucose intake of CAL-51 cells; **D** oxygen consumption rate of CAL-51 cells; **E** ATP level in CAL-51 cells; **F** CAL-51 cell viability; **G** flow cytometry determined cell cycle distribution; **H**, **I** Transwell assay tested migration and invasion ability of CAL-51 cells; **J**, **K** tumor volume and weight of nude mice that had been injected with CAL-51 cells; repetitions = 3 in (**A**–**I**), *n* = 6 in (**J**, **K**); *P < 0.05; **P < 0.01; ***P < 0.001; data of groups were compared using one-way ANOVA, and were expressed as mean ± standard deviation.

After treatment of exosomes, we found that (Fig. 5A, B) in CAL-51 cells, miR-503-3p, active-β-catenin, Glut1, and LDH-A were upregulated while DACT2 and p-β-catenin were downregulated in the exo group versus the PBS group; cells in the miR-503-3p inhibitor-exo group had lower expression levels of miR-503-3p, active-β-catenin, Glut1, and LDH-A, and higher expression levels of DACT2 and p-β-catenin than the exo group (all *P* < 0.05).

Measurement of glucose concentration, oxygen consumption rate, and ATP level showed that (Fig. 5C–E) in CAL-51 cells, glucose intake was enhanced, whereas oxygen consumption rate and ATP level were reduced in the exo group relative to the PBS group; the miR-503-3p inhibitor-exo group had restricted glucose intake and raised oxygen consumption rate and ATP level in relation to the exo group (all *P* < 0.05).

Examinations of cell proliferation (Fig. 5F), cell cycle arrest (Fig. 5G), and the migration and invasion (Fig. 5H, I) found that versus the PBS group, the viability, migration, and invasion of cells were facilitated, and the cell ratio was increased in S phase while decreased in G0/G1 phase in the exo group; compared with the exo group, the viability, migration, and invasion of cells were inhibited, and the cell ratio was decreased in S phase while increased in G0/G1 phase in the miR-503-3p inhibitor-exo group (all *P* < 0.05).

BC cell growth (Fig. 5J, K) showed that versus the PBS group, the tumor weight and volume were increased in the exo group; compared with the exo group, the tumor growth was decreased in the miR-503-3p inhibitor-exo group (*P* < 0.05).

In MDA-MB-231 cells, it was found that versus the PBS and exo group, respectively, the exo group and miR-503-3p mimic-exo group had higher miR-503-3p, active-β-catenin, Glut1, and LDH-A, as well as lower DACT2 and p-β-catenin expression levels (all *P* < 0.05) (Supplementary Fig. 2A, B). Detection of intake of glucose, oxygen consumption rate, and ATP level revealed that versus the PBS group and exo group, respectively, the exo group and miR-503-3p mimic-exo group showed enhanced intake of glucose, reduced oxygen consumption rate and ATP level (all *P* < 0.05) (Supplementary Fig. 2C-E). Observation of cell proliferation, cell cycle, invasion, and migration suggested that with respect to the PBS and exo group, respectively, the cell viability, migration, and invasion rates were facilitated, cell ratio in S phase was increased while that in G0/G1 phase was decreased in the exo group and miR-503-3p mimic-exo group (all *P* < 0.05) (Supplementary Fig. 2F-I). Also, increased tumor volume and weight were detected in the exo group and miR-503-3p mimic-exo group compared with the PBS and exo group, respectively (all *P* < 0.05) (Supplementary Fig. 2J/K).

### Upregulated DACT2 inactivates the Wnt/β-catenin signaling pathway, restrains glycolysis, and elevates mitochondrial OXPHOS in BC cells

**Fig. 6:**
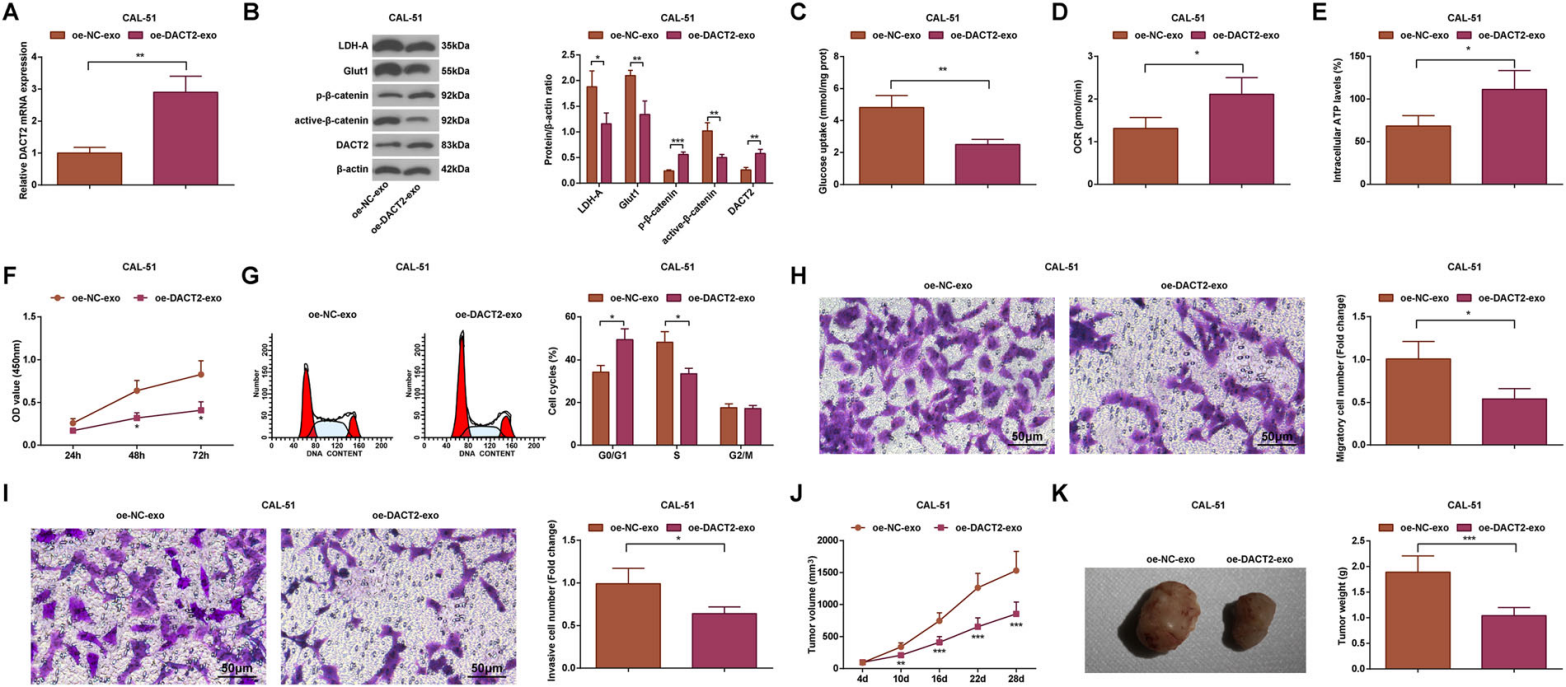
Upregulated DACT2 inactivates the Wnt/β-catenin signaling pathway, restrains glycolysis, and elevates mitochondrial OXPHOS in CAL-51 cells. **A** RT-qPCR measured miR-503-3p and DACT2 expression in CAL-51 cells; **B** western blot analyzed DACT2, active-β-catenin, p-β-catenin, Glut1, and LDH-A protein expression in CAL-51 cells; **C** glucose intake of CAL-51 cells; **D** oxygen consumption rate of CAL-51 cells; **E** ATP level in CAL-51 cells; **F** CAL-51 cell viability; **G** flow cytometry determined cell cycle distribution; **H**, **I** Transwell assay tested migration and invasion ability of CAL-51 cells; **J**, **K** tumor volume and weight of nude mice that had been injected with CAL-51 cells; repetitions = 3 in (**A**–**I**), *n* = 6 in (**J**, **K**); *P < 0.05; **P < 0.01; ***P < 0.001; the measurement data conforming to the normal distribution were expressed as mean ± standard deviation and data of groups were compared using Student’s *t* test.

In exploring the role of DACT2 transferred by macrophage-derived exosomes, it was noticed that (Fig. 6A, B) relative to the oe-NC-exo group, cells in the oe-DACT2-exo group had lower expression of active-β-catenin, Glut1, and LDH-A, and higher expression of DACT2 and p-β-catenin (all *P* < 0.05).

Intake of glucose, oxygen consumption rate, and ATP level was calculated, and the outcomes showed that (Fig. 6C–E) the glucose intake was reduced while oxygen consumption rate and ATP level were enhanced in the oe-NC-exo group relative to the oe-DACT2-exo group (all *P* < 0.05).

Viability, migration, invasion, and cell cycle arrest of BC cells were assessed. It came out that (Fig. 6F–I) relative to the oe-NC-exo group, the cell viability, migration, and invasion rates were inhibited, cell ratio in S phase was decreased while that in G0/G1 phase was increased in the oe-DACT2-exo group (all *P* < 0.05).

Results of subcutaneous tumorigenesis in nude mice revealed that (Fig. 6J, K) in CAL-51 xenografts, the tumor weight and volume were both reduced in the oe-DACT2-exo group versus the oe-NC-exo group (both *P* < 0.05).

In MDA-MB-231 cells, it was found that versus the sh-NC-exo group, the sh-DACT2-exo group had increased active-β-catenin, Glut1, and LDH-A, as well as reduced DACT2 and p-β-catenin expression levels (all *P* < 0.05) (Supplementary Fig. 3A, B). Detection of intake of glucose, oxygen consumption rate, and ATP level revealed that versus the sh-NC-exo group, the sh-DACT2-exo group showed enhanced intake of glucose, reduced oxygen consumption rate, and ATP level (all *P* < 0.05) (Supplementary Fig. 3C-E). Observation of cell proliferation, cell cycle, invasion, and migration suggested that with respect to the sh-NC-exo group, the cell viability, migration, and invasion rates were facilitated, cell ratio in S phase was increased while that in G0/G1 phase was decreased in the sh-DACT2-exo group (all *P* < 0.05) (Supplementary Fig. 3F–I). In xenografted tumors, promoted tumor growth was observed in the sh-DACT2-exo group compared with the sh-NC-exo (both *P* < 0.05) (Supplementary Fig. 3J/K).

## Discussion

BC is the most common cause of cancer death in women and is the 2nd most common cancer death in developed counties after lung cancer^20^. Exosomes, the vital mediators of intercellular communication, have been identified to transfer miRNAs to recipient cells^21^. We performed this research to investigate the impact of macrophage-derived exosomal miR-503-3p in biological processes of BC cells, and it was found that the downregulation of exosomal miR-503-3p restrained glycolysis and promoted mitochondrial OXPHOS in BC cells via upregulating DACT2 and inhibiting the Wnt/β-catenin signaling pathway.

We first assessed expression of miR-503-3p, DACT2, and Wnt/β-catenin signaling pathway-related factors. The outcomes mirrored that miR-503-3p was highly expressed, DACT2 was poorly expressed and the Wnt/β-catenin signaling pathway was activated in BC tissues and cells. The abnormal expression has been clarified in other literatures. For instance, Zhao et al. have suggested that miR-503-3p was upregulated in BC tissues and plasma in relation to adjacent normal breast tissues and plasma from healthy controls^10^. A former document has illustrated that DACT2 was decreased in BC tissues^12^, and it has been revealed that the abnormal activation of Wnt/β-catenin signaling pathway was able to result in many types of cancer, including BC^22^. Moreover, we discovered that the macrophage-derived exosomes elevated the expression of miR-503-3p in BC cells. Similarly, Nguyen et al. have demonstrated that miR-503 was enriched in atherogenic EVs relative to the controls^23^. Another finding in our study indicated that reduced miR-503-3p and elevated DACT2 were able to inactivate the Wnt/β-catenin signaling pathway. In line with this result, it has been illuminated that DACT2 inhibited the Wnt/β-catenin signaling pathway in human BC cells^13^. We also verified the target relation between miR-503-3p and DACT2 in BC. However, this relationship has not been uncovered by now.

BC cells were treated with macrophage-derived exosomes, upregulated/downregulated exosomal miR-503-3p, or silenced/overexpressed DACT2 to investigate the effects of altered miR-503-3p and DACT2 on BC development. Results of our in vitro experiments revealed that the macrophage-derived exosomes, elevation of exosomal miR-503-3p, and silenced DACT2 promoted glycolysis and repressed mitochondrial OXPHOS in BC cells, and also contributed to the malignant behaviors of BC cells. Consistently, Yuan et al. have unraveled that exosomes released from Tat-treated macrophages were able to transfer miR-27a to stimulate glycolysis in lung epithelial cells^24^. A previous research has discovered that cancer-associated fibroblasts-derived exosomes inhibited mitochondrial OXPHOS, thereby promoting glycolysis in cancer cells^25^. In addition, it has been unearthed that tumor-associated macrophages released exosomes to promote invasion of BC cells^26^. For the role of miR-503-3p, Zhao et al. have confirmed that miR-503-3p functioned as an oncogene of BC cell proliferation, migration, and invasion^10^, and it has been recently identified that the amplification of miR-503 promoted growth and inhibited apoptosis of glioblastoma cells^27^. In addition, Li et al. have found that DACT2 suppressed BC cell growth and arrested BC cells at G1/S phase^13^, and it has been unveiled that the overexpression of DACT2 suppressed proliferation and enhanced apoptosis of glioma cells^28^. Furthermore, results of in vivo experiment revealed that macrophage-derived exosomes, elevation of exosomal miR-503-3p, and silenced DACT2 facilitated growth of xenografts. In accordance with this finding, oral squamous cell carcinoma-derived exosomes have been demonstrated to transfer miR-29a-3p to promote tumor growth in nude mice with xenograft^29^, and it has been illuminated that DACT2 decelerated BC cell tumor growth in xenograft mice^13^.

In summary, we found that reduction of macrophage-derived exosomal miR-503-3p repressed glycolysis and promoted mitochondrial OXPHOS in BC cells, and constrained malignant phenotypes of BC cells by overexpressing DACT2 and inactivating the Wnt/β-catenin signaling pathway. This study may further the understanding of molecular mechanisms in BC.

## Supplementary information

Supplementary Figure 1

Supplementary Figure 2

Supplementary Figure 3

supplementary Figure legends
