# Supplementary figures and images for: Exosomal microRNA-503-3p derived from macrophages represses glycolysis and promotes mitochondrial oxidative phosphorylation in breast cancer cells by elevating DACT2
Source: Cell Death Discov. 2021 May 20;7:119. doi: 10.1038/s41420-021-00492-2 (PMC8137952; doi:10.1038/s41420-021-00492-2)

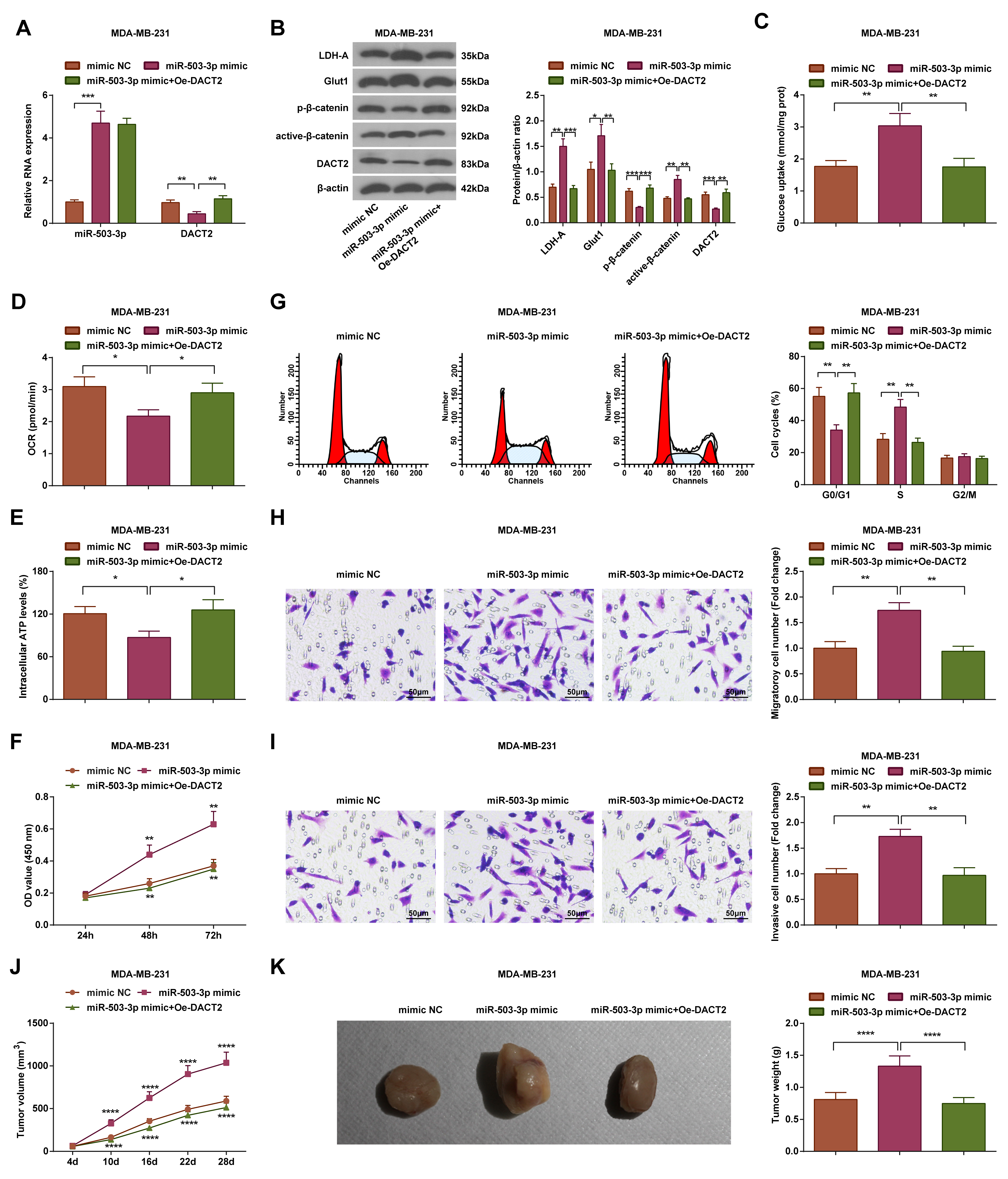

Supplement: Supplementary file 1 — Supplementary Figure 1 [file 41420_2021_492_MOESM1_ESM.tif]

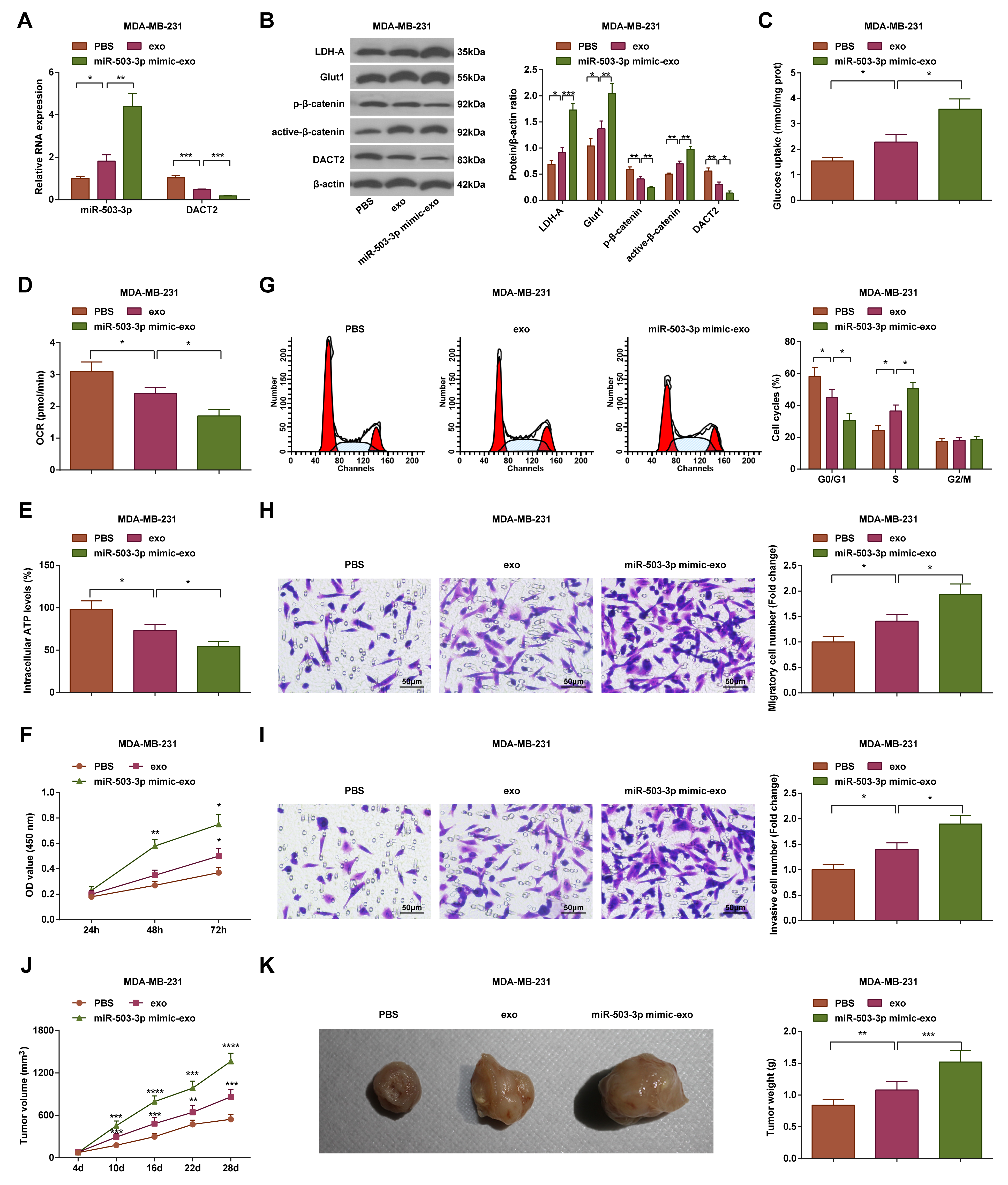

Supplement: Supplementary file 2 — Supplementary Figure 2 [file 41420_2021_492_MOESM2_ESM.tif]

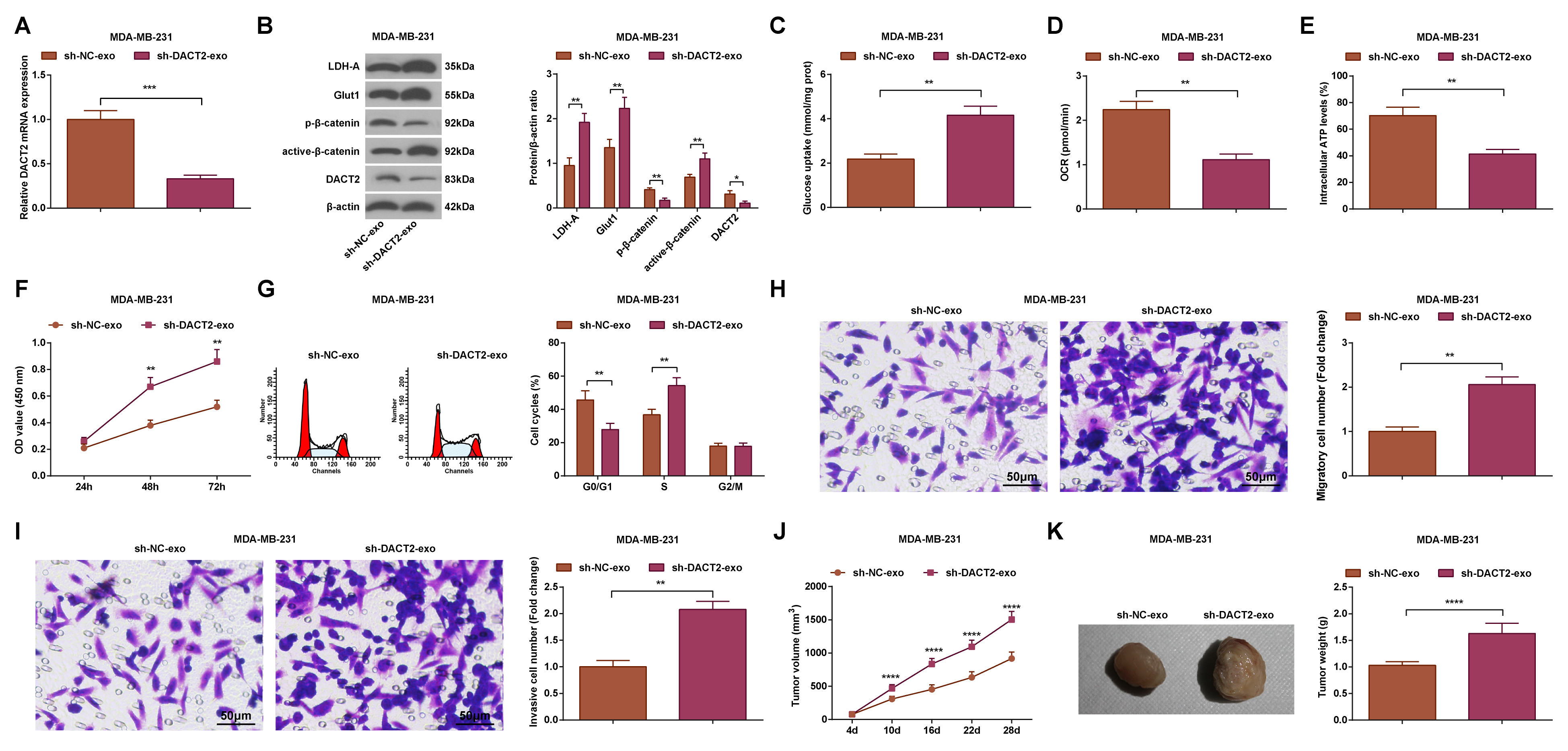

Supplement: Supplementary file 3 — Supplementary Figure 3 [file 41420_2021_492_MOESM3_ESM.tif]
