# Supplementary material for: Exosomal microRNA-503-3p derived from macrophages represses glycolysis and promotes mitochondrial oxidative phosphorylation in breast cancer cells by elevating DACT2
Source: Cell Death Discov. 2021 May 20;7:119. doi: 10.1038/s41420-021-00492-2 (PMC8137952; doi:10.1038/s41420-021-00492-2)
Supplement: Supplementary file 4 — supplementary Figure legends [file 41420_2021_492_MOESM4_ESM.docx]

**Supplementary Figure 1** Upregulation of DACT2 reverses the effect of overexpression of miR-503-3p on MDA-MB-231 cells. A, expression of miR-503-3p and DACT2 in MDA-MB-231 cells was determined using RT-qPCR; B, protein expression of DACT2, active-β-catenin, p-β-catenin, Glut1 and LDH-A in MDA-MB-231 cells was determined using Western blot analysis; C, glucose intake of MDA-MB-231 cells in each group; D, oxygen consumption rate of MDA-MB-231 cells in each group; E, ATP level in MDA-MB-231 cells of each group; F, MDA-MB-231 cell viability in each group; G, cell cycle distribution was determined by flow cytometry; H, migration ability of MDA-MB-231 cells was assessed by Transwell assay; I, invasion ability of MDA-MB-231 cells was assessed by Transwell assay; J, tumor volume of nude mice that had been injected with MDA-MB-231 cells; K, tumor weight of nude mice that had been injected with MDA-MB-231 cells; repetitions = 3 in A-I, n = 6 in J&K;* *P* < 0.05; ** *P* < 0.01; *** *P* < 0.001; **** *P* < 0.0001; the measurement data conforming to the normal distribution were expressed as mean ± standard deviation and data of groups were compared using one-way ANOVA.

**Supplementary Figure 2** Macrophage-derived exosomal miR-503-3p activates the Wnt/β-catenin signaling pathway, promotes glycolysis and reduces mitochondrial OXPHOS in MDA-MB-231 cells. A, expression of miR-503-3p and DACT2 in MDA-MB-231 cells was determined using RT-qPCR; B, protein expression of DACT2, active-β-catenin, p-β-catenin, Glut1 and LDH-A in MDA-MB-231 cells was determined using Western blot analysis; C, glucose intake of MDA-MB-231 cells in each group; D, oxygen consumption rate of MDA-MB-231 cells in each group; E, ATP level in MDA-MB-231 cells of each group; F, MDA-MB-231 cell viability in each group; G, cell cycle distribution was determined by flow cytometry; H, migration ability of MDA-MB-231 cells was assessed by Transwell assay; I, invasion ability of MDA-MB-231 cells was assessed by Transwell assay; J, tumor volume of nude mice that had been injected with MDA-MB-231 cells; K, tumor weight of nude mice that had been injected with MDA-MB-231 cells; repetitions = 3 in A-I, n = 6 in J&K; * *P* < 0.05; ** *P* < 0.01; *** *P* < 0.001; **** *P* < 0.0001; data of groups were compared using one-way ANOVA, and were expressed as mean ± standard deviation.

**Supplementary Figure 3** Macrophage-derived exosomes transfer of down-regulated DACT2 activates the Wnt/β-catenin signaling pathway, promotes glycolysis and reduces mitochondrial OXPHOS in MDA-MB-231 cells. A, expression of DACT2 in MDA-MB-231 cells was determined using RT-qPCR; B, protein expression of DACT2, active-β-catenin, p-β-catenin, Glut1 and LDH-A in MDA-MB-231 cells was determined using Western blot analysis; C, glucose intake of BC cells in each group; D, oxygen consumption rate of MDA-MB-231 cells in each group; E, ATP level in MDA-MB-231 cells of each group; F, MDA-MB-231 cell viability in each group; G, cell cycle distribution was determined by flow cytometry; H, migration ability of MDA-MB-231 cells was assessed by Transwell assay; I, invasion ability of MDA-MB-231 cells was assessed by Transwell assay; J, tumor volume of nude mice that had been injected with MDA-MB-231 cells; K, tumor weight of nude mice that had been injected with MDA-MB-231 cells; repetitions = 3 in A-I, n = 6 in J&K; * *P* < 0.05; ** *P* < 0.01; *** *P* < 0.001; **** *P* < 0.0001; the measurement data conforming to the normal distribution were expressed as mean ± standard deviation and data of groups were compared using Student’s t test.
